# Targeted Quantitative Metabolomics and Lipidomics Reveal Dysregulated Metabolic Networks and a Serum Candidate Biomarker for Atrial Fibrillation

**DOI:** 10.3390/metabo16090630

**Published:** 2026-08-29

**Authors:** Yuqing Zhang, Yunpeng Xie, Xinyu Liu, Zhen Ning, Guowang Xu, Yunlong Xia, Xinjie Zhao

**Affiliations:** 1 Metabolomics Subcenter of the National Genomics Data Center, Dalian Institute of Chemical Physics, Chinese Academy of Sciences, Dalian 116023, China; yuqingzhang@dicp.ac.cn (Y.Z.); liuxy2012@dicp.ac.cn (X.L.); xugw@dicp.ac.cn (G.X.); 2 School of Chemistry, Dalian University of Technology, Dalian 116024, China; 3 The First Affiliated Hospital of Dalian Medical University, Dalian 116011, China; xieyunpeng@dmu.edu.cn (Y.X.); ningzhen@dmu.edu.cn (Z.N.); 4 University of Chinese Academy of Sciences, Beijing 100049, China; 5 Liaoning Province Key Laboratory of Metabolomics, Dalian 116023, China

**Keywords:** metabolomics, lipidomics, atrial fibrillation, serum biomarker, metabolic network

## Abstract

Background: Atrial fibrillation (AF) is the most prevalent clinical arrhythmia with severe cardiovascular complications, yet its metabolic molecular mechanisms remain poorly defined. Omics-based metabolic profiling provides a powerful strategy to systematically decode AF-associated metabolic disorders. Methods: In this work, high-coverage targeted liquid chromatography–tandem mass spectrometry (LC-MS/MS) metabolomics and lipidomics were applied to absolutely quantify 746 serum metabolites from AF patients and healthy controls. Results: We systematically characterized global metabolic perturbations in AF serum, including impaired fatty acid metabolism, suppressed mitochondrial β-oxidation, myocardial lipotoxic lipid accumulation, and systemic depletion of glycerophospholipids. Global multiscale embedded correlation network analysis (MECNA) further identified 11 AF-specific dysregulated metabolic modules and core hub metabolites driving metabolic remodeling. Leveraging binary logistic regression, we constructed and independently validated a two-molecule diagnostic biomarker panel to distinguish AF patients from healthy subjects. The combined biomarkers Phe-Trp and FA 22:5 achieved outstanding diagnostic performance, with area under the curve (AUC) values of 0.964 in the discovery cohort and 0.993 in the validation cohort. Conclusions: Collectively, this study adopts high-depth targeted quantitative omics to comprehensively map AF metabolic signatures, dissect disease-relevant metabolic networks, and establish a robust serum biomarker panel with great translational potential for non-invasive AF clinical diagnosis.

## 1. Introduction

Atrial fibrillation (AF) represents the most prevalent sustained cardiac arrhythmia in clinical practice [1]. The global prevalence of atrial fibrillation had reached 52.55 million individuals in 2021, causing a substantial cardiovascular disease burden worldwide [2]. As a progressive disorder with intricate pathological mechanisms, AF confers markedly elevated risks of severe cardiovascular complications including thromboembolism, ischemic stroke and heart failure, which contribute to increased disability and mortality among affected patients [3,4,5]. To date, several circulating biomarkers, such as N-terminal pro-B-type natriuretic peptide, cardiac troponin and C-reactive protein, have been routinely implemented for clinical risk stratification and disease evaluation of AF [6,7,8,9]. Nevertheless, the molecular mechanisms underlying the onset and progression of AF remain incompletely elucidated. Accordingly, comprehensive characterization of AF-related metabolic perturbations via innovative approaches beyond conventional clinical biochemistry is urgently warranted.

Metabolomics and lipidomics are powerful omics strategies that profile global metabolic signatures, providing critical insights into biomarker screening and metabolic pathway exploration in cardiovascular diseases [10]. Previous studies have preliminarily explored the metabolic characteristics of AF using mass spectrometry-based metabolomics. Smith et al. identified 112 fasting plasma metabolites via LC-MS and verified that seven medium- and long-chain acylcarnitines were closely correlated with elevated AF risk [11]. In another LC-MS-based study focusing on plasma polar metabolites, Razquin et al. reported that higher baseline kynurenine levels were significantly associated with increased AF susceptibility [12]. Lu et al. combined gas chromatography–mass spectrometry (GC-MS) and LC-MS to quantify 211 metabolites and screened out 25 potential AF-related metabolic biomarkers [13]. Lai et al. also constructed a combined plasma biomarker signature consisting of D-glutamic acid, creatinine, and choline, which achieved a high diagnostic accuracy for AF with an AUC of 0.927 [14]. However, existing metabolite biomarker identification and validation predominantly rely on untargeted metabolomics approaches. The absence of absolute quantitative data severely compromises the accuracy and reproducibility of these biomarkers, greatly restricting their translational application in clinical practice.

Our group established a robust alternating two-dimensional liquid chromatography coupled with triple quadrupole mass spectrometry (LC-MS/MS) strategy operating in multiple reaction monitoring (MRM) mode [15,16]. This high-coverage platform enables high-throughput absolute quantification of more than 1000 metabolites and lipids with diverse physicochemical properties, covering amino acids, acylcarnitines, glycerolipids, phospholipids, and other key metabolic categories. Compared with untargeted approaches that only generate relative quantitative data, this targeted workflow achieves precise absolute quantification with excellent sensitivity, repeatability, and stability, as validated by low relative standard deviations of most analytes. Benefiting from expanded metabolite coverage and standardized quantitative procedures, this advanced technique effectively eliminates the data inconsistency and poor reproducibility of traditional untargeted metabolomics, providing a reliable technical basis for rigorous AF biomarker screening, validation, and subsequent clinical translation.

**Figure 1:**
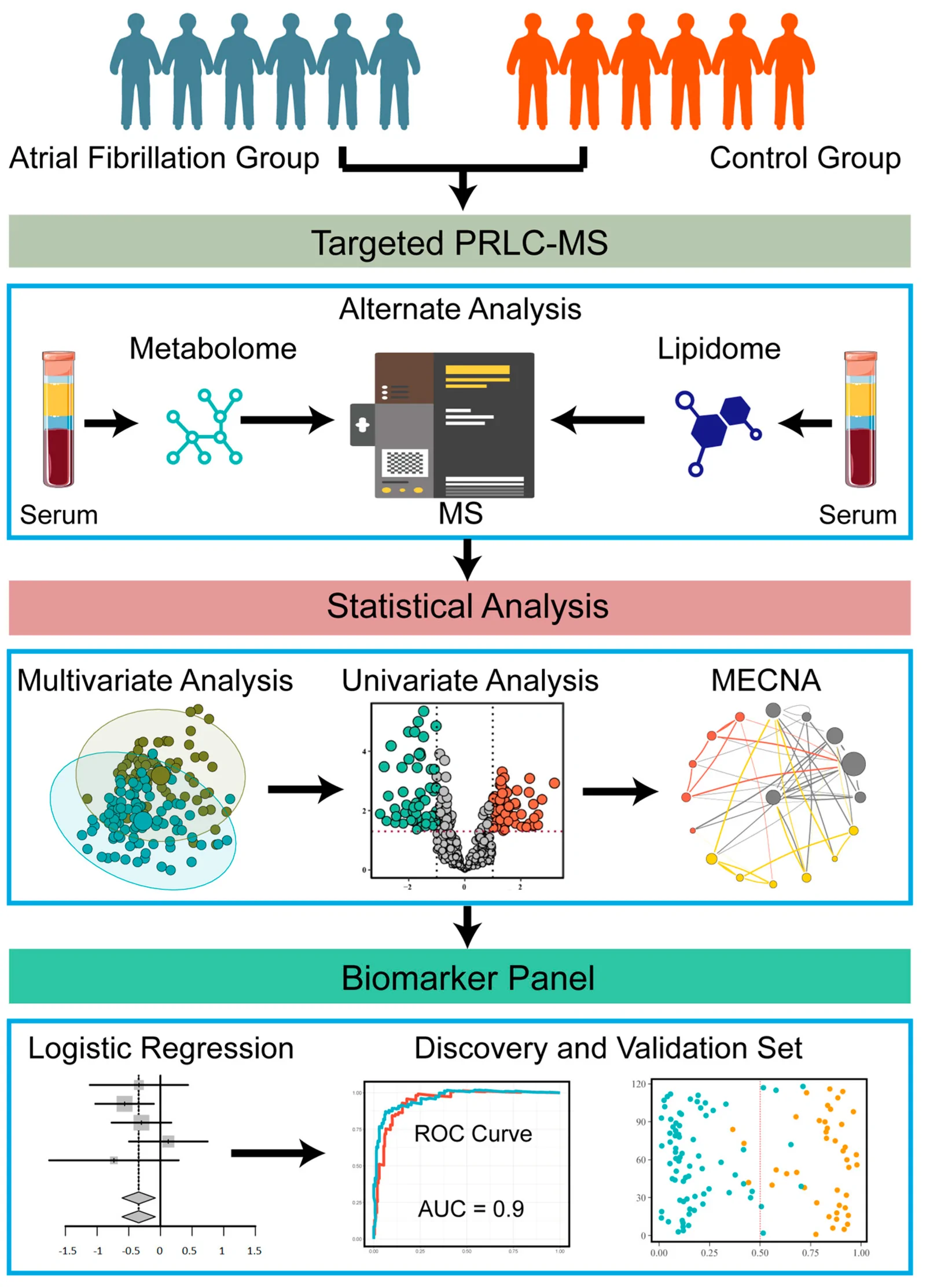
Schematic workflow of targeted serum metabolomic and lipidomic profiling for atrial fibrillation (AF) research. Serum samples were collected from AF patients and healthy controls and analyzed via alternating reversed-phase liquid chromatography–mass spectrometry (RPLC-MS/MS). Multivariate statistics, univariate analysis, and multiscale embedded correlation network analysis (MECNA) were performed to screen differential metabolites and core hub molecules. Binary logistic regression was applied to construct and validate a two-molecule diagnostic biomarker panel with receiver operating characteristic (ROC) curve evaluation.

In this study, we applied high-coverage targeted LC-MS-based metabolomics and lipidomics to perform absolute quantification of serum metabolites based on two independent cohorts comprising 261 participants. Benefiting from expanded metabolite coverage and standardized quantitative procedures, this advanced technique effectively eliminates the data inconsistency and poor reproducibility of traditional untargeted metabolomics. We first characterized the distinct metabolic signatures between AF patients and healthy controls. Dysregulated AF-associated metabolic networks were further constructed via metabolite enrichment and correlation network analysis (MECNA) to identify core regulatory metabolite hubs. Moreover, a concise two-metabolite biomarker panel was screened and validated using binary logistic regression, which exhibited robust diagnostic performance for AF. Collectively, this study aims to systematically elucidate the metabolic reprogramming underlying AF pathogenesis and develop a quantitative serum biomarker panel to support auxiliary clinical diagnosis of AF. The overall research workflow is illustrated in Figure 1.

## 2. Materials and Methods

### 2.1. Chemicals and Reagents

The ultrapure water used in this experiment was produced by the Milli-Q system (Millipore, Billerica, MA, USA). The HPLC-grade acetonitrile (ACN), methanol (MeOH), and isopropanol (IPA) were purchased from Merck (Darmstadt, Germany). Ammonium acetate (AmAc) and ammonium bicarbonate (NH_4_HCO_3_) were purchased from Fluka (St. Louis, MO, USA) and Sigma-Aldrich (St. Louis, MO, USA), respectively. Formic acid (FA) was acquired from J&K Scientific (Beijing, China). Methyl-tert-butyl ether (MTBE) were from Aladdin (Shanghai, China).

### 2.2. Sample Information

A total of 261 participants, including individuals with AF and healthy controls, were recruited from the First and Second Affiliated Hospitals of Dalian Medical University for this study. AF was diagnosed according to 12-lead ECG and/or ambulatory Holter monitoring following clinical guidelines. The participants were divided into discovery and validation cohorts. The clinical information of participants in the discovery and validation cohorts was listed in Table 1. The study has received official approval from the Clinical Ethics Committee of the First Affiliated Hospital and the Second Affiliated Hospital of Dalian Medical University, and strictly adheres to the ethical guidelines established by the 1964 Declaration of Helsinki. Written informed consent was obtained from all participants.

### 2.3. Sample Pretreatment and RPLC-MS Analyses

The pretreatment procedures for serum samples followed the methods described in previous published studies of this group [15]. Metabolome extraction process: 200 µL MeOH containing metabolome internal standards was added to 50 µL serum sample, vortexed for 2 min, and centrifuged (18,700 *g*, 10 °C, 15 min). Then, 100 µL of supernatant was freeze-dried overnight. The dried samples were redissolved in 150 µL ACN/H_2_O (2:8, *v*/*v*) and 50 µL MeOH/H_2_O (2:8, *v*/*v*) solvents before positive and negative ion mode analyses for the metabolome.

Lipidome extraction process: 300 µL MeOH containing lipidome internal standards was added to 40 µL serum sample and vortexed for 1 min. Then, 1 mL MTBE was added to the mixture and vortexed for 15 min. Next, 300 µL H_2_O was added and vortexed for 30 s. The mixture was then kept at 4 °C for 10 min and centrifuged (18,700 *g*, 10 °C, 15 min). Finally, 400 µL of the upper-layer organic phase was freeze-dried overnight. The freeze-dried samples were redissolved in 640 µL and 120 µL ACN/IPA/H_2_O (65:30:5 *v*/*v*/*v*) solvents containing 5 mM AmAc prior to positive and negative ion mode analyses for the lipidome. The information on internal standards for the metabolome and lipidome is listed in Table S1.

In this study, we used an alternating RPLC separation technique (Nexera X2 systems, Kyoto, Japan) combined with a triple quadrupole mass spectrometry system manufactured by AB SCIEX (Framingham, MA, USA) to achieve a high-coverage analysis of the metabolome and lipidome profiles of biological samples. In positive and negative ion modes, chromatographic columns BEH C8 (2.1 mm × 50 mm, 1.7 µm, Waters, Milford, MA, USA) and Acquity HSS T3 (2.1 mm × 50 mm, 1.8 µm, Waters, Milford, MA, USA) were used for metabolome analysis, respectively. The chromatographic column used for detection in the positive and negative ion modes of the lipidome was BEH C8 (2.1 mm × 100 mm, 1.7 µm, Waters, Milford, MA, USA). The column temperature for the metabolome and lipidome was set at 60 °C. The autosampler temperature of the metabolome and lipidome injection rooms was 4 °C and 10 °C, respectively. The flow rates of the metabolome and lipidome groups were 0.4 mL min^−1^ and 0.3 mL min^−1^, respectively. The detailed information on LC separations is listed in Table S2. The experimental parameters of mass spectrum data acquisition were set according to our previously published work [15]; detailed MRM parameters are provided in Supplementary Tables S3 and S4. In this study, all samples were analyzed in a randomized order. In addition, to monitor the stability of the analysis process, after analyzing 10 samples to be tested, we introduced one QC sample and one blank sample.

### 2.4. Data Processing and Statistical Analysis

Raw LC-MS peak areas of metabolites and lipids were integrated using SCIEX OS v3.0 software. Linear regression calibration curves with a weighting factor of 1/x^2^ were generated for absolute quantification, and all metabolite/lipid signals were normalized to corresponding internal standard peak areas for batch correction. Metabolites and lipids were excluded from downstream analysis if their detection rate across all study samples was below 85% or relative standard deviation (RSD) in quality control (QC) samples exceeded 30%.

Unsupervised principal component analysis (PCA) was performed via SIMCA-P 13.0 to visualize global metabolic separation between groups. Student’s t-test and fold change calculation were performed using the MetaboAnalyst 5.0 platform (www.metaboanalyst.ca). Multivariate linear regression was adjusted for confounding covariates including recruitment hospital, sex, age, systolic blood pressure (SBP), diastolic blood pressure (DBP), uric acid (UA), fasting blood glucose (FPG), creatinine (Cr), total cholesterol (TC), and triglycerides (TG). The R package MECNA v4.0.4 was adopted for multiscale embedded correlation network analysis, with the minimum module size set to 10 and Pearson correlation coefficients used for correlation calculation (significance threshold α = 0.05) [17]. Network visualization was completed with Cytoscape v3.8.0.

Differentially abundant metabolites and core network hub nodes were incorporated into a binary logistic regression model for biomarker screening. All metabolite concentrations were Z-score normalized (mean = 0, variance = 1) before model construction.

## 3. Results

### 3.1. Comprehensive Characterization of the Serum Metabolome and Lipidome

**Figure 2:**
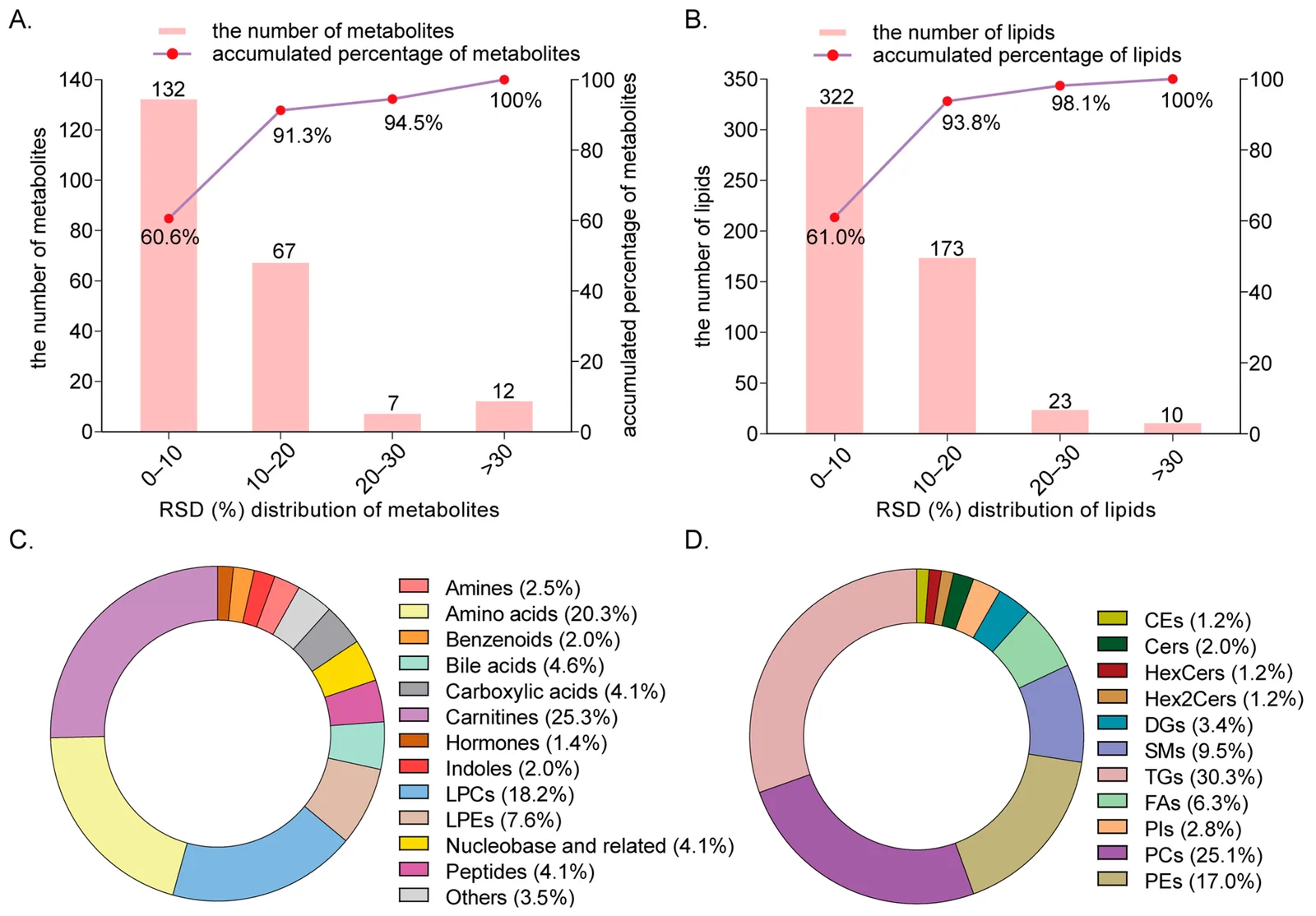
Data quality control and classification distribution of serum metabolites and lipids. (**A**,**B**) Relative standard deviation (RSD) distribution of 218 metabolites and 528 lipids from quality control (QC) samples to assess analytical stability. (**C**,**D**) Pie charts illustrating the subclasses and proportional distribution of qualified metabolites and lipids after filtering. Abbreviations: LPCs, lysophosphatidylcholines; LPEs, lysophosphatidylethanolamines; CEs, cholesterol esters; Cers, ceramides; HexCers, exosylceramides; Hex2Cers, dihexaglycosylceramides; DGs, diacylglycerols; SMs, sphingomyelins; TGs, triacylglycerols; FAs, fatty acids; PIs, phosphatidylinositols; PCs, phosphatidylcholines; PEs, phosphatidylethanolamines.

In this study, serum metabolic profiles of all participants were comprehensively characterized using targeted metabolomics and lipidomics approaches based on an alternating injection dual reversed-phase liquid chromatography coupled with mass spectrometry (2 × RPLC-MS) strategy. In total, 218 metabolites and 528 lipids were initially identified and quantified across QC samples. To guarantee the reliability and reproducibility of quantitative data, relative standard deviation (RSD) values derived from QC samples were adopted to evaluate analytical stability. The results demonstrated that 94.5% of metabolites and 98.1% of lipids had RSD values below 30%, indicating satisfactory analytical precision (Figure 2A,B). Additionally, metabolites and lipids with a detection rate lower than 85% in all tested samples were excluded to eliminate unreliable analytical variables. Accordingly, nine metabolites and 12 lipids were discarded prior to subsequent statistical analysis. After strict data quality filtering, a final panel consisting of 197 metabolites and 506 lipids was retained for downstream bioinformatic analysis. The detailed classification and proportional distribution of the qualified metabolites and lipids are presented in Figure 2C,D.

### 3.2. Dysregulated Metabolites and Lipids in Serum of AF Patients

**Figure 3:**
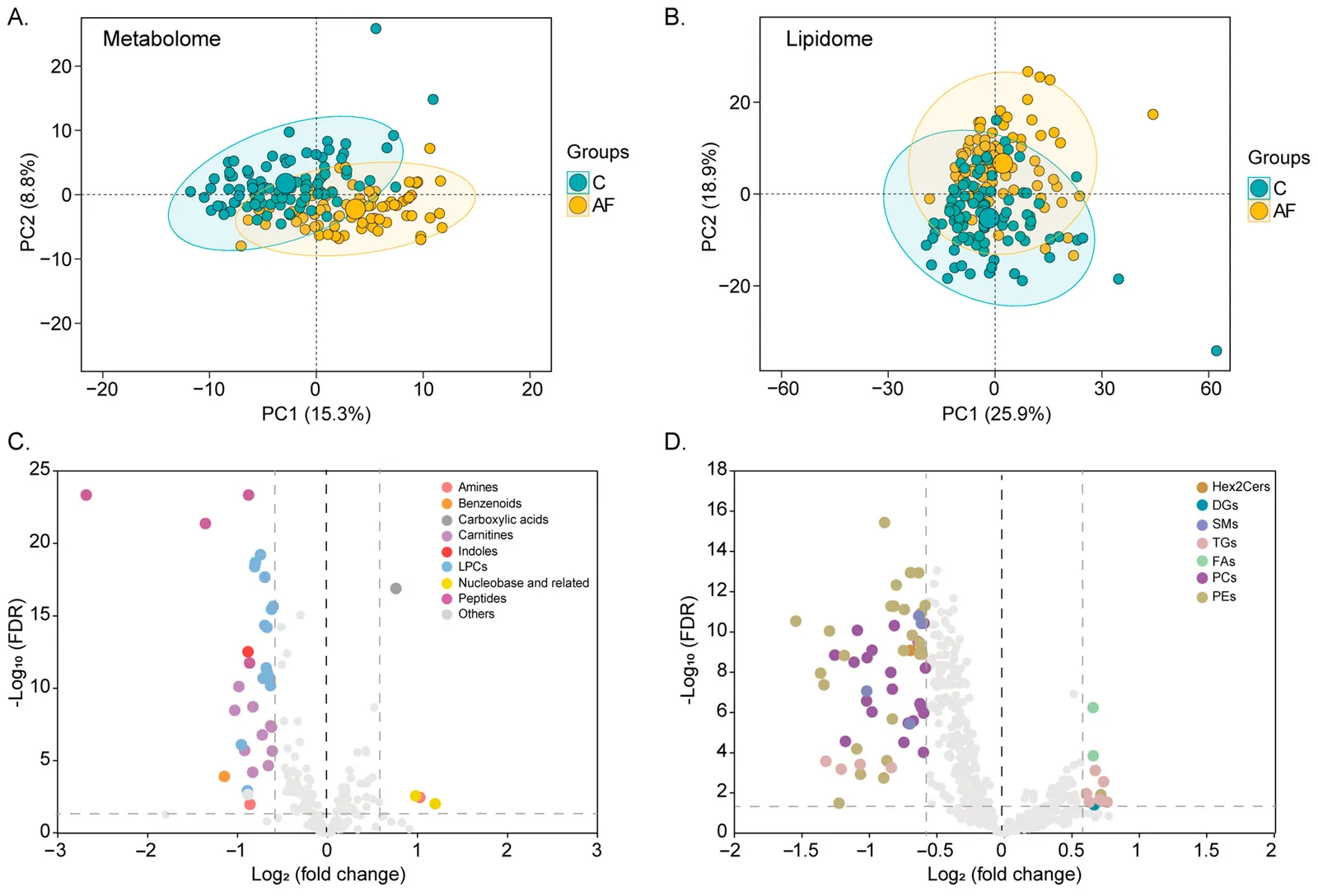
Global metabolic alterations in serum from AF patients versus healthy controls. (**A,B**) PCA score plots of metabolome and lipidome datasets showing distinct separation between AF and control cohorts. (**C,D**) Volcano plots displaying differential metabolites and lipids. Vertical dashed lines mark log_2_(FC) corresponding to fold-change thresholds of 0.67 (downregulated) and 1.5 (upregulated), and the horizontal dashed line denotes −log_10_(FDR) = 2 (FDR = 0.01). Molecules with false discovery rate (FDR) < 0.05 and fold change (FC) > 1.5 (upregulated) or FC < 0.67 (downregulated) are highlighted in colored dots.

To characterize AF-specific metabolic perturbations, targeted metabolomic and lipidomic profiling was performed on serum samples from 97 patients with confirmed AF and 76 healthy controls. Principal component analysis (PCA) of both metabolome and lipidome datasets revealed a distinct separation trend between the AF patients and healthy controls, suggesting substantially altered serum metabolic profiles in AF patients (Figure 3A,B). Based on univariate statistical analysis, we observed a total of 105 significantly differential metabolites and lipids(FDR < 0.05, FC > 1.5). Specifically, 14 metabolites and lipids were significantly upregulated (FC > 1.5), whereas 91 metabolites and lipids were downregulated (FC < 0.67) in the AF patients relative to healthy controls (Table S3, Figure 3C,D).

Four dipeptides (Asp-Phe, Gly-Val, Phe-Phe, and Phe-Trp) and multiple acylcarnitines showed significant decreases in AF patients. In addition, major glycerophospholipid subclasses, including LPCs, PCs, and PEs, were broadly decreased in AF patients. In contrast, two long-chain polyunsaturated fatty acids, FA 18:3 and FA 22:5, were significantly accumulated in AF patients. For neutral lipids, the diacylglycerol DG(16:0_16:0) was distinctly elevated in AF patients. In addition, triglyceride species exhibited unsaturation-dependent differential alterations: TGs with low double-bond numbers (1–8) were generally increased, while highly unsaturated TGs (11–14 double bonds) were decreased in AF serum.

In summary, high-coverage metabolomics and lipidomics results demonstrate widespread metabolic changes in AF patients. The distinct alterations in amino acid dipeptides, acylcarnitines, glycerophospholipids, fatty acids, and triglycerides reflect disrupted metabolic homeostasis, providing important metabolic evidence for the pathophysiological characteristics of AF.

### 3.3. Multiscale Embedded Correlation Network to Reveal Dysregulated Modules Associated with AF

**Figure 4:**
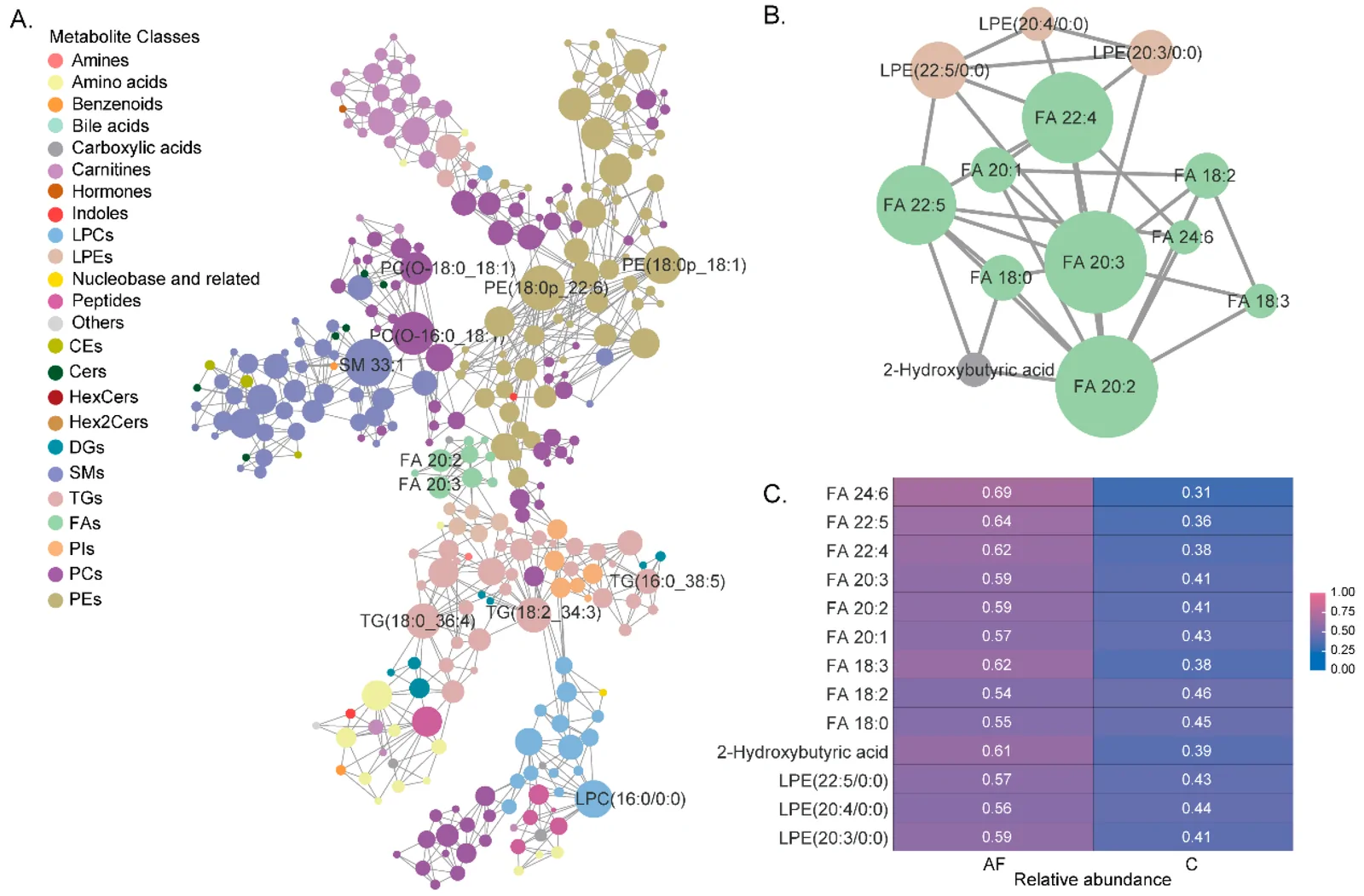
Multiscale embedded correlation network analysis (MECNA) of AF-associated differential metabolites and lipids. (**A**) Global correlation network constructed from 312 significantly AF-related molecules, with nodes colored according to metabolite/lipid subclasses and key hub metabolites labeled. (**B**) Subnetwork centered on core hub fatty acids FA 20:3 and FA 20:2. (**C**) Heatmap showing relative serum abundance of molecules in the FA-centered subnetwork between AF patients and healthy controls.

To explore the molecular network modules driving AF-related metabolic dysfunction, a covariate-adjusted linear regression model was applied to screen metabolites and lipids significantly correlated with AF. After adjusting for multiple confounding factors, including sampling location, sex, age, systolic blood pressure, diastolic blood pressure, uric acid, fasting plasma glucose, creatinine, total cholesterol, and triglycerides, a total of 312 AF-associated metabolic molecules were identified(adjusted *p* < 0.05) (Table S4). All qualified AF-related metabolites and lipids were used to calculate pairwise correlations, based on which a metabolic correlation network was constructed. MECNA was applied to partition the network into modules, and functional enrichment analysis was conducted on all metabolites within each module. In total, 11 distinct dysregulated metabolic modules were determined to be closely involved in AF pathogenesis (Figure 4A). Metabolites and lipids within the same chemical category exhibited obvious aggregation in the network, indicating consistent structural similarity and potential synergistic co-regulatory relationships during AF metabolic perturbation. Further module-focused analysis prioritized long-chain fatty acids as key AF-correlated metabolic components based on regression results (Table S4). Subnetwork visualization further constructed long-chain fatty acid-centered modules, with FA 20:3 and FA 20:2 identified as the core hub metabolites (Figure 4B). Corresponding heatmap profiling demonstrated that metabolites within this fatty acid-centered subnetwork were significantly upregulated in AF patients relative to healthy controls (Figure 4C). In addition, multiple independent dysregulated metabolic modules were also successfully identified in the constructed network, with TGs, PEs, PCs, SMs, and LPCs as core hubs.

### 3.4. Biomarker Panel Derived from Logistic Regression Model Enables AF Patient Diagnosis

In this study, metabolites exhibiting significant changes (FC > 1.5 or FC < 0.67, and adj. *p* < 0.05), as well as core hub metabolites screened via MECNA network analysis, were integrated into a binary logistic regression model to screen diagnostic signatures that discriminate AF patients from healthy controls. We performed feature selection using a binary logistic regression model based on the discovery cohort. The selected metabolites were further applied to construct a binary logistic regression classifier. Finally, a dual-molecule panel consisting of Phe-Trp and FA 22:5 was established, which exhibited robust diagnostic performance for distinguishing atrial fibrillation. Odds ratio (OR) analysis revealed that Phe-Trp was negatively associated with AF risk (OR = 0.037), whereas FA 22:5 was positively correlated with elevated AF susceptibility (OR = 7.226).

**Figure 5:**
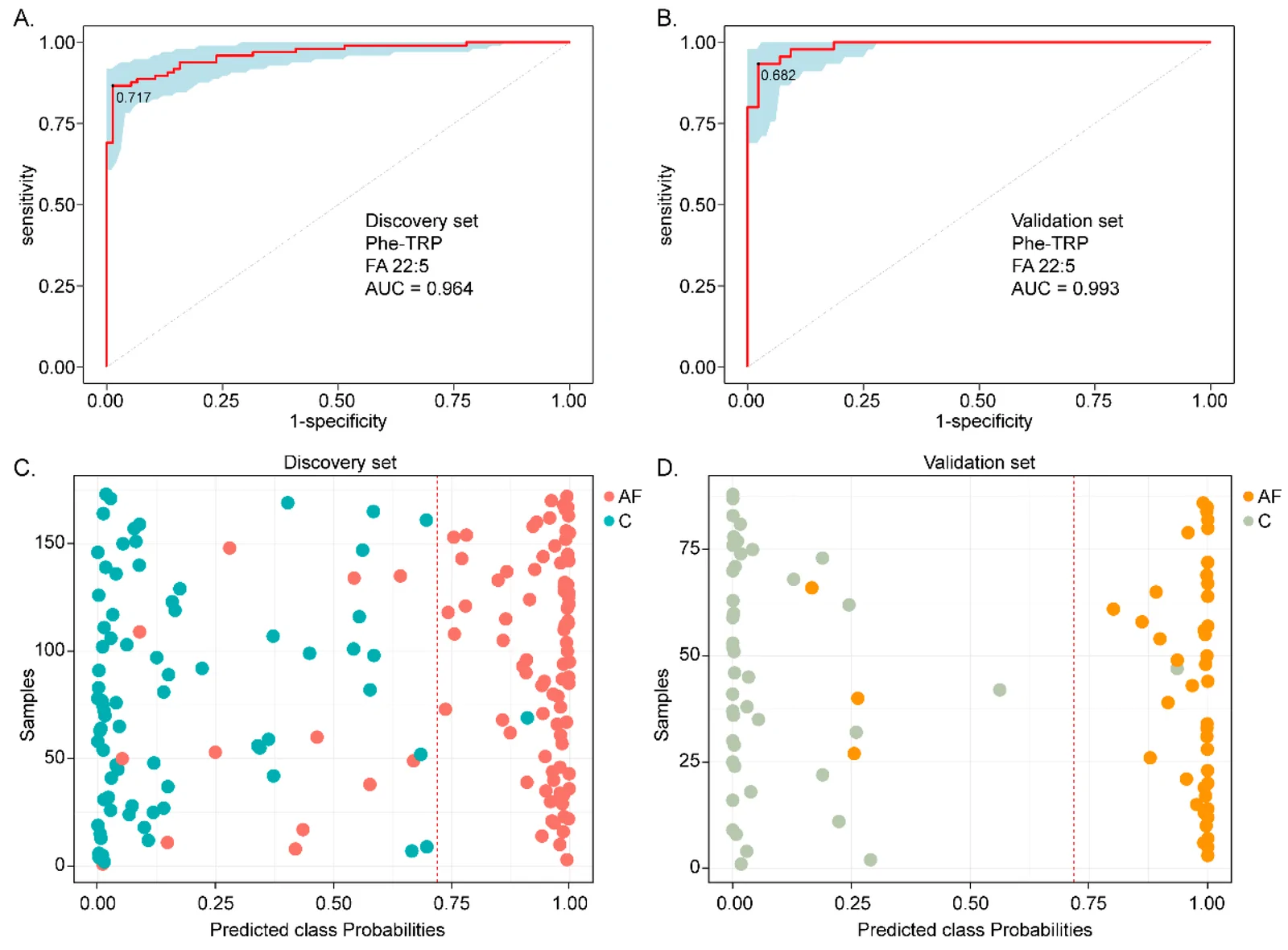
Diagnostic performance of the dual-molecule biomarker panel (Phe-Trp + FA 22:5) constructed via binary logistic regression. (**A**,**B**) ROC curves and corresponding area under the curve (AUC) values in the discovery cohort and independent validation cohort. (**C**,**D**) Scatter plots displaying predicted AF probabilities of all participants in discovery and validation cohorts based on the logistic regression model. Vertical red dashed lines indicate the cutoff obtained for group classification.

Subsequently, the established classifier was executed on a validation cohort to assess the generalizability and independence of the model. In the discovery cohort and validation cohort, the area under the curve (AUC) was 0.964 (specificity 85.5%, sensitivity 90.7%) and 0.993 (specificity 95.3%, sensitivity 93.3%), respectively, as shown in Figure 5A,B. The diagnostic equation based on the concentrations of Phe-Trp and FA 22:5 in serum is constructed as follows:

$$logit\left[P=AF\right]=1.978\times \left[FA\;22:5\right]-3.288\times \left[Phe-Trp\right]+0.595$$

where [P = AF] represents the predictive probability of the combined biomarkers for AF; [FA 22:5] is the concentration of FA 22:5 in serum, measured in µM; and [Phe-T rp] is the concentration of Phe-Trp in serum, also measured in µM. The threshold for [P = AF] in the discovery cohort was found to be 0.717. With a cut-off value of 0.717 for classification, the prediction accuracy of the model is 92.5% and 95.1% in the discovery and validation cohorts (Figure 5C,D). In the discovery cohort, the false positive rate was 1%, and the false negative rate was 12%; in the validation cohort, the false positive rate was 2%, and the false negative rate was 7%.

Collectively, these findings highlight the powerful capacity of integrated metabolomic and lipidomic profiling for the discovery and validation of AF-specific biomarkers. The dual-metabolite signature developed herein provides a novel quantitative serum tool for auxiliary AF diagnosis, which may advance precise clinical surveillance and management of AF patients.

## 4. Discussion

AF ranks as the most prevalent sustained cardiac arrhythmia encountered in routine clinical care [18]. Metabolomics and lipidomics have emerged as powerful platforms to unravel the molecular mechanisms driving AF pathogenesis and can further advance the development of innovative diagnostic strategies for this disease [19,20]. In the study, our alternating injection strategy integrates targeted metabolomic and lipidomic profiling for accurate absolute quantification of up to 746 molecular species covering central carbon metabolism, amino acids, and diverse lipid subclasses. This high-coverage, quantitative omics strategy allows us to capture coordinated cross-talk between polar metabolite and lipid perturbations that jointly underpin AF metabolic remodeling.

The present metabolomic and lipidomic analysis revealed profound and multilayered serum metabolic dysregulation in patients with atrial fibrillation (AF), primarily involving perturbed amino acid metabolism, impaired fatty acid oxidation, altered glycerophospholipid homeostasis, and myocardial lipotoxicity. At the metabolite subclass level, AF patients exhibited significant depletion of circulating dipeptides and acylcarnitines. As key intermediate products of protein degradation and amino acid recycling, four representative dipeptides (Asp-Phe, Gly-Val, Phe-Phe, and Phe-Trp) were markedly reduced in AF serum. Prior research demonstrates that proteolytic degradation of atrial sarcomeric proteins constitutes the mechanistic basis of contractile impairment in atrial fibrillation [21]. Combined with our concurrent findings of decreased acylcarnitines and impaired fatty acid oxidation, the loss of circulating dipeptides further demonstrates the existence of a severe myocardial energy crisis in AF.

Fatty acid oxidation (FAO) is the predominant source of myocardial energy supply, meeting approximately 70% of the basal energy requirements of the adult heart [22]. The mitochondrial β-oxidation of medium- and long-chain fatty acids is strictly dependent on the carnitine transport system, in which carnitine palmitoyltransferase 1 (CPT1) mediates the binding and mitochondrial translocation of cytoplasmic fatty acids, thereby enabling subsequent lipid catabolism [23]. Under AF conditions, atrial energy demand rises drastically, prompting a compensatory metabolic shift toward glucose and ketone utilization to offset energy shortages [24,25]. Consistent with previous studies identifying disrupted fatty acid metabolism in AF [26], our results revealed a distinct metabolic disorder characterized by simultaneous serum acylcarnitine depletion and long-chain fatty acid accumulation in AF patients. The reduced abundance of acylcarnitines directly indicates dysfunctional carnitine-dependent fatty acid transport, which impedes mitochondrial β-oxidation and blocks the normal breakdown of long-chain fatty acids. This effective uncoupling of the carnitine transport system and downstream FAO results in impaired lipid energy metabolism and persistent myocardial energy insufficiency, which is a central pathogenic factor driving atrial structural and electrical remodeling in AF [23,26].

In addition, we observed prominent decreases in multiple glycerophospholipid subclasses, including LPCs, PCs, and PEs, in AF serum. As essential structural components of cell membranes and key regulators of inflammatory responses, the global depletion of glycerophospholipids implies dysregulated activity of cytosolic phospholipase A2 (PLA2), a critical enzyme responsible for phospholipid hydrolysis [27]. Large-scale cohort studies have validated that aberrant Lp-PLA2 activity is independently associated with elevated incident AF risk, driving persistent myocardial inflammatory injury and adverse atrial remodeling [28]. Collectively, PLA2 family dysfunction contributes to progressive membrane damage, chronic low-grade inflammation, and maladaptive cardiac hypertrophy, which collectively exacerbate atrial microstructural injury and arrhythmia progression. Notably, two long-chain polyunsaturated fatty acids (FA 18:3 and FA 22:5) were significantly upregulated in AF patients, likely representing a compensatory antioxidant response. However, excessive polyunsaturated fatty acid accumulation may trigger lipid peroxidation and aggravate atrial oxidative damage, forming a detrimental feedback loop that sustains AF progression.

Importantly, our results identified typical metabolic signatures of cardiac lipotoxicity in AF patients. Lipotoxicity is characterized by aberrant myocardial lipid deposition, which induces mitochondrial dysfunction, endoplasmic reticulum stress, cardiomyocyte apoptosis, and electrical instability [29,30]. In this study, lowly unsaturated TGs (1–8 double bonds) and DG(16:0_16:0) were significantly elevated in AF serum (FC > 1.5), which are well-recognized biomarkers of lipotoxic lipid accumulation [31]. Mechanistically, accumulated DGs act as second messengers to activate protein kinase C (PKC) signaling, which directly triggers myocardial electrical disturbance and arrhythmogenesis [32]. Collectively, these findings demonstrate that integrated disturbances in amino acid, fatty acid, and glycerophospholipid metabolism drive energy deficiency, oxidative stress, inflammation, and lipotoxicity, ultimately facilitating the occurrence and progression of AF.

MECNA further reveals the interrelated metabolic modules that are disturbed in atrial fibrillation. These modules mirror coordinated metabolic reprogramming, integrating impaired protein turnover, mitochondrial fatty-acid catabolism defects, membrane lipid breakdown, and lipotoxic stress into a unified pathological cascade driving atrial remodeling.

Blood biomarkers can effectively guide disease diagnosis and clinical monitoring. In clinical practice, 12-lead electrocardiogram and ambulatory Holter monitoring have always been regarded as the “gold standard” for AF diagnosis. However, this AF screening method is primarily based on detecting the onset period of the disease, which may result in an inability to effectively identify paroxysmal AF. Metabolomic biomarkers provide an overall overview of the body’s systems, informing us about the changes that have occurred in AF conditions. We use a binary logistic regression model to identify and validate a biomarker panel (Phe-Trp and FA 22:5) related to atrial fibrillation. Decreased circulating Phe-Trp reflects impaired atrial protein degradation and myocardial energy deficiency [21], whereas elevated FA 22:5 corresponds to dysregulated polyunsaturated fatty acid metabolism with dual roles in antioxidant compensation and pro-oxidative lipid peroxidation [33]. The combination of these two molecules integrates signatures of myocardial protein catabolism and lipid-mediated oxidative stress, two core pathological features of AF. We investigate metabolic abnormalities and identify biomarkers through omics approaches can provide a perspective beyond clinical biochemistry and electrophysiology for understanding the molecular mechanisms of AF, while also offering new clues for its diagnosis and clinical management. However, the current sample size is limited and insufficient for conducting stratified analysis of clinical subtypes of atrial fibrillation. In the future, it is necessary to construct a large stratified cohort with complete clinical phenotype information to further validate the stability and translational value of this serum marker in different subgroups of atrial fibrillation.

## 5. Conclusions

In the present study, high-coverage absolute quantitative targeted LC-MS metabolomics and lipidomics were integrated to systematically dissect serum metabolic reprogramming in patients with atrial fibrillation (AF). Comprehensive univariate and multivariate statistical analyses revealed broad metabolic disturbances centered on impaired mitochondrial fatty acid β-oxidation, systemic depletion of glycerophospholipids, and abnormal accumulation of lipotoxic lipids, which jointly drive atrial energy deficiency, inflammation, and electrical remodeling. Multiscale embedded correlation network analysis (MECNA) further resolved 11 distinct AF-associated dysregulated metabolic modules, with long-chain polyunsaturated fatty acids identified as core hub metabolites governing metabolic disorder. After binary logistic regression feature screening and independent two-cohort validation, we constructed a robust two-molecule serum biomarker panel combining Phe-Trp and FA 22:5, which achieves outstanding diagnostic accuracy for non-invasive AF identification. Collectively, this research expands our mechanistic insights into AF metabolic pathogenesis and delivers a reliable quantitative serum omics signature that holds great translational value for auxiliary clinical diagnosis and precise risk stratification of atrial fibrillation in routine cardiovascular practice.

## Figures and Tables

**Table 1:** Baseline clinical characteristics of participants in the discovery cohort and independent validation cohort.

	Discovery Cohort		Validation Cohort	
Characteristic	Healthy Controls	AF Patients	Healthy Controls	AF Patients
N	76	97	43	45
Female	47 (61.84%)	41 (42.27%)	23 (53.49%)	19 (42.22%)
Male	29 (38.16%)	56 (57.73%)	20 (46.51%)	26 (57.78%)
Age (years) (±SD)	50.5 ± 7.80	63.18 ± 9.68	47.14 ± 7.94	62.51 ± 9.37
SBP (mmHg) (±SD)	120.75 ± 10.63	128.3 ± 16.01	121.21 ± 10.50	130.36 ± 14.48
DBP (mmHg) (±SD)	74.32 ± 8.42	79.84 ± 11.36	75.40 ± 8.47	81.98 ± 11.12
UA (µmol/L) (±SD)	360.62 ± 105.56	339.55 ± 90.46	388.36 ± 104.89	364.47 ± 87.64
BG (mmol/L) (±SD)	5.32 ± 0.37	5.45 ± 1.42	5.34 ± 0.40	5.25 ± 1.25
Cr (µmol/L) (±SD)	66.61 ± 14.21	69.65 ± 17.23	69.10 ± 15.84	76.77 ± 19.82
TC (mmol/L) (±SD)	5.12 ± 1.08	4.59 ± 1.08	5.22 ± 0.82	4.26 ± 0.83
TG (mmol/L) (±SD)	1.49 ± 0.73	1.47 ± 0.70	1.60 ± 0.90	1.36 ± 0.62

## Data Availability

Data will be made available upon request from the corresponding authors. The data are not publicly available due to privacy concerns.

## Supplementary Materials

The following supporting information can be downloaded at https://www.mdpi.com/article/10.3390/metabo16090630/s1. Table S1. Detailed information on internal standards applied for metabolome and lipidome absolute quantification; Table S2. Liquid chromatography separation parameters for targeted metabolomic and lipidomic detection; Table S3. Information on metabolomics MRM transitions and method validation results of selected analytes; Table S4. Information on lipidomics MRM transitions and method validation results of selected lipids; Table S5. Significantly differential serum metabolites and lipids between atrial fibrillation (AF) patients and healthy controls (FDR < 0.05, FC > 1.5 or FC < 0.67); Table S6: Metabolites and lipids significantly correlated with AF after adjustment for multiple clinical confounding factors.
